# SiO_2 _nanoparticles induce cytotoxicity and protein expression alteration in HaCaT cells

**DOI:** 10.1186/1743-8977-7-1

**Published:** 2010-01-19

**Authors:** Xifei Yang, Jianjun Liu, Haowei He, Li Zhou, Chunmei Gong, Xiaomei Wang, Lingqing Yang, Jianhui Yuan, Haiyan Huang, Lianhua He, Bing Zhang, Zhixiong Zhuang

**Affiliations:** 1 Key Laboratory of Modern Toxicology of Shenzhen, Shenzhen Centre for Disease Control and Prevention, No. 21, Road 1st Tianbei, Luohu District, Shenzhen, 518020, PR China; 2 Department of Biochemistry and Molecular Biology, Life Science School, Shenzhen University, Nanhai Ave 3688, Shenzhen, 518060, PR China

## Abstract

**Background:**

Nanometer silicon dioxide (nano-SiO_2_) has a wide variety of applications in material sciences, engineering and medicine; however, the potential cell biological and proteomic effects of nano-SiO_2 _exposure and the toxic mechanisms remain far from clear.

**Results:**

Here, we evaluated the effects of amorphous nano-SiO_2 _(15-nm, 30-nm SiO_2_). on cellular viability, cell cycle, apoptosis and protein expression in HaCaT cells by using biochemical and morphological analysis, two-dimensional differential gel electrophoresis (2D-DIGE) as well as mass spectrometry (MS). We found that the cellular viability of HaCaT cells was significantly decreased in a dose-dependent manner after the treatment of nano-SiO_2 _and micro-sized SiO_2 _particles. The IC_50 _value (50% concentration of inhibition) was associated with the size of SiO_2 _particles. Exposure to nano-SiO_2 _and micro-sized SiO_2 _particles also induced apoptosis in HaCaT cells in a dose-dependent manner. Furthermore, the smaller SiO_2 _particle size was, the higher apoptotic rate the cells underwent. The proteomic analysis revealed that 16 differentially expressed proteins were induced by SiO_2 _exposure, and that the expression levels of the differentially expressed proteins were associated with the particle size. The 16 proteins were identified by MALDI-TOF-TOF-MS analysis and could be classified into 5 categories according to their functions. They include oxidative stress-associated proteins; cytoskeleton-associated proteins; molecular chaperones; energy metabolism-associated proteins; apoptosis and tumor-associated proteins.

**Conclusions:**

These results showed that nano-SiO_2 _exposure exerted toxic effects and altered protein expression in HaCaT cells. The data indicated the alterations of the proteins, such as the proteins associated with oxidative stress and apoptosis, could be involved in the toxic mechanisms of nano-SiO_2 _exposure.

## Background

With the rapid development of nanotechnology and its applications, nano-structured materials have been widely used in the fields of biomedicine, pharmaceutical, and other industrial business. Nanometer silicon dioxide (nano-SiO_2_) is one of the most popular nanomaterials that are being used in these fields such as industrial manufacturing, packaging, high-molecule composite materials and ceramics synthesis, disease labeling, drug delivery, cancer therapy and biosensor. Nano-SiO_2 _particles can be readily evaporated into air due to their very low density. Inhalation of SiO_2 _nanoparticles causes pulmonary and cardiovascular alterations and damages in old rats, such as pulmonary inflammation, myocardial ischemic damage, atrio-ventricular blockage, and increase in fibrinogen concentration and blood viscosity [1]. Nano-SiO_2 _exposure also results in DNA damage [2], size-dependent hydroxyl radicals generation [3] and lung fibrogenesis in rats [4]. Skin is a potential primary route of occupational dermal exposure for nanometer materials. Due to the difficulty for macrophages to efficiently scavenge nanoparticles in the skin [5], the potential toxicological effects of nano-SiO_2 _exposure will be probably caused in the skin. However, little is known about the potential dermal toxicity of nano-SiO_2 _exposure, and the molecular basis of nano-SiO_2 _toxicity in the dermal cells. The present study was undertaken to explore the effects of manufactured nano-SiO_2 _particles on cellular viability, cell cycle, apoptosis as well as protein expression in human epidermal keratinocyte cell line HaCaT.

## Results

### 1. Characterization of SiO_2 _particles

**Figure 1:**
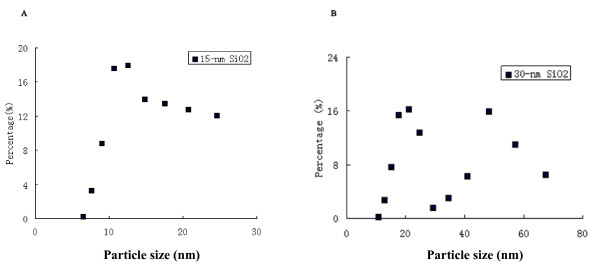
**The distribution of the three types of SiO_2 _particles**. The particle size distribution of 15-nm and 30-nm SiO_2 _was shown in A and B, respectively.

The results from characterization of 15-nm, 30-nm and micro-sized SiO_2 _were summarized in Table 1. The average diameter of the particles in 15-nm SiO_2 _sample was 13.0 ± 1.8 nm, accounting of almost 100% of all the particles in the sample. The 30-nm SiO_2 _sample were mainly composed of the particles with average diameters of 20.1 ± 3.5 nm (50.5%) and 51.3 ± 9.2 nm (49.5%), respectively. The micro-sized SiO_2 _sample consisted mostly of particles with average diameters of 365.1 ± 79.5 nm, accounting of almost 100% of all the particles in the sample. The particle size distribution of nano-SiO_2 _particles was shown in Figure 1. Since the size of most of the particles exceeded 100 nm; this sample could be considered as the control of micro-sized SiO_2 _particles.

**Table 1:** Characterization of 15-nm, 30-nm and micro-sized SiO_2_

	Size and distribution nm (mean ± SD)	Zeta potential (mV)	Crystalline structure
15-nm SiO_2_	13.0 ± 3.8 nm	-14.37	Amorphous
30-nm SiO_2_	20.1 ± 3.5 nm (50.5%); 51.3 ± 9.2 nm (49.5%)	-63.31	Amorphous
Micro-sized SiO_2_	365.1 ± 79.5 nm	-59.7	Amorphous

Zeta potential of SiO_2 _particles, a parameter of particle diffusion degree, was measured. The zeta potential of 15-nm, 30-nm and micro-sized SiO_2 _particles was -14.37 mV, -63.31 mV and -59.70 mV, respectively (Table 1). These results indicated that 15-nm SiO_2 _particle was relatively less stable than 30-nm and micro-sized SiO_2 _particles. The purity testing showed that the purity of all the 3 types of SiO_2 _particles was higher than 99.7%. A small quantity of sodium but no heavy metals were detected among the 3 types of samples (data not shown). X ray diffraction (XRD) analysis revealed that the structure of 15-nm, 30-nm SiO_2 _and micro-sized SiO_2 _particles was amorphous (Table 1).

### 2. The toxicological effects of nano-SiO_2 _exposure on HaCaT cells

#### 2.1. The morphological effects of nano-SiO_2 _exposure on HaCaT cells

**Figure 2:**
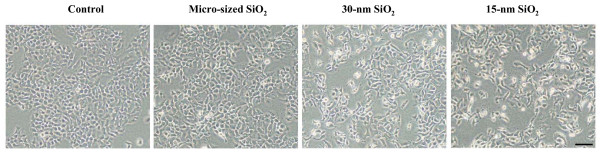
**Morphological changes induced by SiO_2 _exposure**. HaCaT cells were exposed to 15-nm, 30-nm and micro-sized SiO_2 _particles at 10 μg/mL for 24 h. Scale bar = 30 μm.

Morphological examination revealed that after 24-h exposure of HaCaT cells to 15-nm, 30-nm and micro-sized SiO_2 _particles at 10 μg/mL, the cell growth was significantly inhibited, and some cells became irregular shapes, dead and floated. The dead cells became floated above the living cells. The morphological changes were more obvious in smaller (15-nm and 30-nm) SiO_2 _particle-treated cells than the larger (micro-sized) SiO_2 _particle -treated cells (Figure 2).

#### 2.2. The effects of nano-SiO_2 _exposure on cellular viability of HaCaT cells

**Figure 3:**
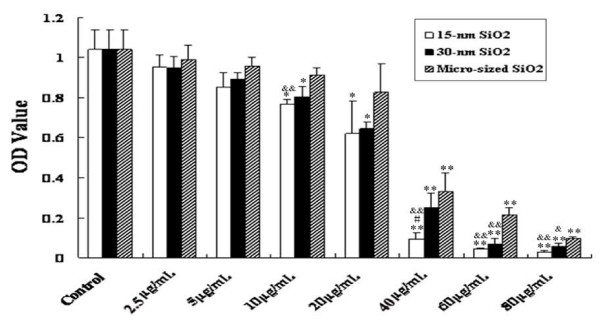
**Viability of HaCaT cells after 24-h exposure to 15-nm, 30-nm or micro-sized SiO_2 _particles**. Values were mean ± SD from three independent experiments. *p < 0.05, **p < 0.01 vs control cells; ^#^*p *< 0.05 vs cells exposed to 30-nm SiO_2 _particles; ^&^*p *< 0.05, ^&&^*p *< 0.01 vs cells exposed to micro-sized SiO_2 _particles.

Exposure of HaCaT cells to 15-nm, 30-nm and micro-sized SiO_2 _particles resulted in significantly decreased cell viability in a dose-dependent manner (Figure 3). Also, HaCaT cells exposed to smaller size (15-nm) SiO_2_particles at the same dosage higher than 40 μg/mL exhibited lower cell viability than the ones exposed to the larger ones (30-nm and micro-sized SiO_2 _particles, Figure 3). In general, the smaller the particles were, the lower the cell viability was. The relationship of inhibitory rate of the cell growth and the dosages was analyzed by SPSS 13.0 software, and the IC_50 _(50% concentration of inhibition) value was therefore figured out. The IC_50 _value of 15-nm, 30-nm and micro-sized SiO_2 _particles was 23.0 μg/mL, 27.3 μg/mL and 34.8 μg/mL, respectively. The dosage of 15 μg/mL (about 1/2-2/3 IC_50 _for 15-nm SiO_2_) was used as the maximum dosage in this study, and 2.5 μg/mL, 5 μg/mL and 10 μg/mL were selected as the final dosages used in the subsequent experiments.

#### 2.3. The effects of nano-SiO_2 _exposure on cell cycle of HaCaT cells

**Figure 4:**
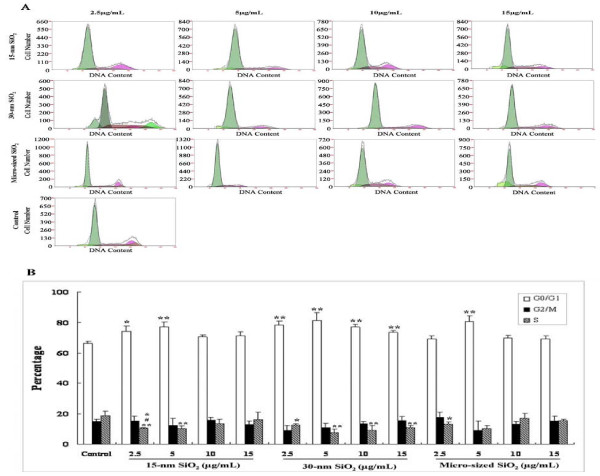
**The effects of exposure to 15-nm, 30-nm SiO_2 _and micro-sized SiO_2 _particles on cell cycle of HaCaT cells**. A shows the representative images of cell cycle of HaCaT cells after 24-h exposure to 15-nm, 30-nm or micro-sized SiO_2_particles at the dosages of 2.5 μg/mL, 5 μg/mL, 10 μg/mL or 15 μg/mL. B shows the results of the quantitative cell cycle analysis. Values were mean ± SD from three independent experiments. *p < 0.05, **p < 0.01 vs control cells; ^#^*p *< 0.05 vs cells exposed to 30-nm SiO_2 _particles; ^&^*p *< 0.05 vs cells exposed to micro-sized SiO_2 _particles.

Flow cytometric analysis of cell cycle showed that exposure to 15-nm, 30-nm and micro-sized SiO_2 _particles could induce G arrest in HaCaT cells as evidenced by increased ratio of the cells at G0/G1 (Figure 4A, 4B). In addition, compared to the control, the ratio of the cells at S phase was significantly reduced in the cells exposed to 15-nm, 30-nm and micro-sized SiO_2 _particles at different dosages. No significant dose- or size-effect relationships were observed.

#### 2.4. The effects of nano-SiO_2 _exposure on apoptosis of HaCaT cells

**Figure 5:**
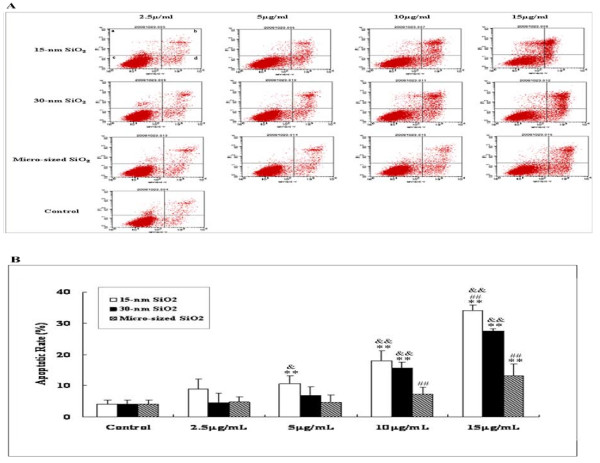
**The effects of exposure to 15-nm, 30 nm or micro-sized SiO_2 _particles onon cellular apoptosis of HaCaT cells**. A shows the representative images of apoptosis of HaCaT cells after 24-h exposure to 15-nm, 30-nm or micro-sized SiO_2 _particles at the dosages of 2.5 μg/mL, 5 μg/mL, 10 μg/mL or 15 μg/mL. B shows the quantitative results. Quadrant a, b, c and d denote the ratio of necrotic cells, late-stage apoptotic cells, normal cells, and early-stage apoptotic cells, respectively. Values were mean ± SD from three independent experiments. **p < 0.01 vs control cells; ^##^*p *< 0.01 vs cells exposed to 30-nm SiO_2 _particles; ^&^*p *< 0.05, ^&&^*p *< 0.01 vs cells exposed to micro-sized SiO_2 _particles.

Flow cytometric analysis showed that exposure to 15-nm, 30-nm or micro-sized SiO_2 _particles induced apoptosis in HaCaT cells in a dose-dependent manner (Figure 5A, 5B). The apoptotic rate in 15-nm SiO_2_-treated cells was significantly higher than that of 30-nm or micro-sized SiO_2_-treated cells (*p *< 0.05 or 0.01, Figure 5B). The apoptotic rate induced by exposure to 30-nm SiO_2 _particles at the dosage of 10 μg/mL or 15 μg/mL was significantly higher than that one induced by micro-sized SiO_2_particles (*p *< 0.01, Figure 5B). Taken together, these data indicated that at the same dosages (for middle and high dosages), the smaller SiO_2 _particles were, the severer apoptosis the cells underwent.

### 3. The effects of nano-SiO_2 _exposure on protein expression in HaCaT cells

#### 3.1. 2D-DIGE analysis

**Figure 6:**
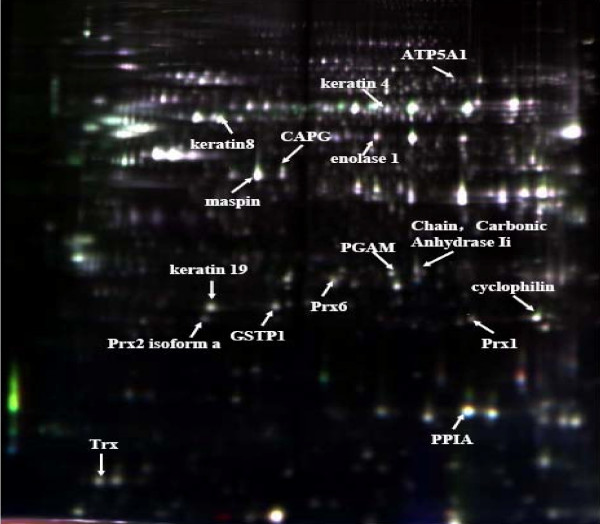
**A representative 2D-DIGE image of the whole lysate of HaCaT cells exposed to 15-nm, 30-nm or micro-sized SiO_2 _particles at 10 μg/mL**. The denoted protein spots were those altered by SiO_2 _exposure and identified by MS. The differentially expressed proteins were quantitatively analyzed and were listed in Table 2 along with protein identifiers.

To detect and identify the altered proteins induced by nano-SiO_2 _exposure, 2D-DIGE was carried out. Approximately 1 mg total proteins of nano-SiO_2 _or micro-sized SiO_2_-treated HaCaT cells were applied to 2D-DIGE. The gel was used as reference gel stained with coomassie and used for spot cutting and protein sequencing. The whole procedure was repeated three times. Well-resolved, reproducible patterns of total protein expression profiles were obtained. The representative raw gel image illustrated the differentially expressed protein spots due to SiO_2 _exposure at 10 μg/mL, which were identified by mass spectrometry (MS) (Figure 6)

#### 3.2. Identification of differentially expressed proteins by mass spectrometry (MS)

**Figure 7:**
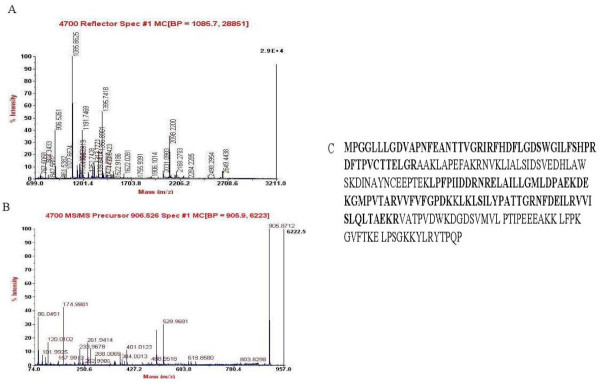
**The MALDI-TOF-MS map of Prx6 was shown in (A)**. B displayed the MS/MS spectra of peptide m/z 906.53 of Prx6, C displayed the amino acid sequences of Prx6 in which matched peptide sequence was in bold, and the sequence coverage was 58%.

**Figure 8:**
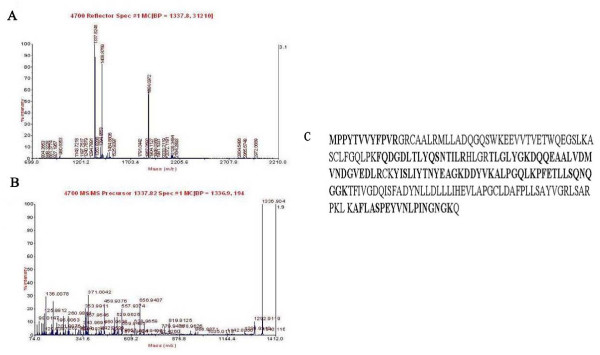
**The MALDI-TOF-MS map of GSTP1 was shown in (A)**. B displayed the MS/MS spectra of peptide m/z 1337.82 of GSTP1, C displayed the amino acid sequences of GSTP1 in which matched peptide sequence was in bold, and the sequence coverage was 52%.

MALDI-TOF-MS/MS analysis resulted in identification of 16 protein spots, which were listed in Table 2. The exemplary data of MS/MS of peroxiredoxin (Prx) 6 and glutathione S-transferase pi 1 (GSTP1) were presented in Figure 7 and 8. The 16 proteins could be generally classified into 5 categories (Table 2). They included oxidative stress-associated proteins; cytoskeleton-associated proteins; molecular chaperones; energy metabolism-associated proteins; apoptosis and tumor-associated proteins.

**Table 2:** The fold change and MS results of the differently expressed proteins

**Spot no**.	Protein ID	Fold change (dosage:10 μg/mL)			**NCBI database no**.	Mr (Da)/PI	Protein score	Coverage rate* %
						
		Micro-sized SiO_2_	30-nm SiO_2_	15-nm SiO_2_				
**Oxidative stress-associated proteins**								
717	peroxiredoxin 6	-1.21	-.199	-2.11	gi|4758638	25019.2/6	413	58
796	peroxiredoxin 1	-1.20	-1.44	-1.63	gi|4505591	22096.3/8.2	397	54
806	peroxiredoxin 2 isoform a	-1.33	-1.62	-1.88	gi|32189392	21878.2/5.66	375	47
1019	thioredoxin	-1.22	-2.59	-2.44	gi|50592994	11729.7/4.82	161	45
779	GSTP1	-1.31	-1.84	-1.95	gi|4699783	23325/4.55	253	52
**Cytoskeleton-associated proteins**								
416	gelsolin-like capping protein (CAPG)	-1.46	-1.92	-2	gi|63252913	38474.5/5.82	168	29
248	keratin 8	-1.37	-1.31	-1.35	gi|33875698	55787.2/5.62	640	53
760	keratin 19	1.89	4.35	4.06	gi|34783124	45587/5.11	402	51
217	keratin 4 (408 AA)	--	1.31	1.20	gi|34073	57249.8/6.25	289	44
**Molecular chaperones**								
797	cyclophilin	-1.45	-1.90	-1.87	gi|4758950	23727.5/9.42	310	67
922	PPIA	--	-1.45	-1.37	gi|48145531	17986.9/7.68	71	44
**Metabolism-associated proteins**								
307	enolase 1 variant	-1.30	-1.92	-2.17	gi|62896593	47111.3/7.01	265	50
688	phosphoglycerate mutase (PGAM)	-1.21	-1.86	-1.79	gi|89035672	28831.8/6.67	240	43
132	ATP5A1	-1.27	-1.79	-1.79	gi|4757810	59713.6/9.16	444	50
682	Chain, CCarbonic Anhydrase Ii	-1.48	-2.34	-2.36	gi|443135	28718.7/6.63	338	59
**Apoptosis and tumor-associated proteins**								
436	maspin	-1.36	-1.60	-1.70	gi|4505789	42111.4/5.72	219	33

### 4. Western-blot analysis of Prx 6 and GSTP1

**Figure 9:**
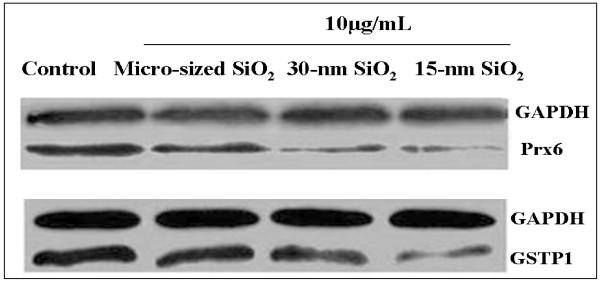
**The changes of Prx6 and GSTP expression induced by SiO_2 _exposure Western-blot showed the change of Prx6 and GSTP1**. The representative bands from three independent experiments were shown.

In order to verify the data from proteomic analysis, we measured the levels of oxidative stress-related proteins, Prx6 and GSTP1, by Western-blot analysis with the specific antibodies (Figure 9). Western-blot results showed that compared to the control, the levels of Prx6 and GSTP1 were significantly decreased in 15-nm, 30-nm or micro-sized SiO_2_-treated HaCaT cells, further validating the proteomic results.

## Discussion

In this study, we explored the potential dermal toxicity of nano-SiO_2 _exposure. We found that nano-SiO_2 _exposure significantly decreased cell viability in a size- and dose-dependent manner. Cell cycle change and apoptosis were also induced by the exposure. The effects of micro-sized SiO_2 _particles on these changes were much milder than that of nano-SiO_2 _particles, indicating that nanoparticles had stronger toxic effects than traditional particles. The proteomic analysis revealed that 16 differentially expressed proteins were induced by SiO_2 _particles in HaCaT cells. The identified 16 proteins by MALDI-TOF-MS/MS were invloved in oxidative stress, cytoskeleton, molecular chaperone, metabolism, apoptosis and tumor.

### Characterization of SiO_2 _particles

The interaction between SiO_2 _nanoparticles and biological system depends on particle diameter, zeta potential and chemical purity. We characterized these properties of three different SiO_2 _used in this study. The results showed that the particle sizes of 15-nm, 30-nm and micro-sized SiO_2 _particles were consistent with the ones provided by the manufacturer. Micro-sized SiO_2 _particles (>100 nm) were used as the control in this study. The data on zeta potential indicated that 15-nm SiO_2 _was relatively less stable than 30-nm and micro-sized SiO_2 _particles. The results from the high purity analysis indicated that the potential toxic effects of other chemical components such as heavy metals could be excluded in this study. The characteristic analysis of the SiO_2_particles showed that the manufactured nano-SiO_2 _and micro-sized SiO_2 _particles used in the present study were stable and reliable for the experiments.

### The toxicity of SiO_2 _nanoparticles

Increasing lines of evidence indicate that many nanomaterials have potential toxicity due to their unique physical-chemical properties [6]. HaCaT is an immortalized epithelial cell line from adult human skin that exhibits similar biological properties to normal human keratinocyte, and is an ideal cell model for studying dermal toxicity [7]. The effects of SiO_2 _nanoparticles on cellular viability, cell cycle as well as apoptosis in HaCaT cells were tested. The data showed that nano-SiO_2 _and micro-sized SiO_2 _exposure could significantly decreased cell viability, cell cycle change, and that the cell viability was closely related to the particle size. Moreover, exposure to SiO_2 _particles also induced apoptosis with a size- and dose-dependent manner. The apoptotic rate was higher in small SiO_2 _particle (15-nm)-treated cells than the large SiO_2 _particle (30-nm and micro-sized)-treated cells. These results demonstrated SiO_2 _particle exposure produced obvious toxicological effects. Previous studies showed that the active dot in the surface of SiO_2 _nanoparticles could react with oxygen molecules, and produce superoxide and other reactive oxygen species (ROS) through disproportionated reaction [8,9]. ROS, resulting in protein and DNA oxidative damage, were therefore likely to be involved in the induction of the toxic effects of SiO_2 _particles as observed. However, the information on how SiO_2 _particles react with cells remains unclear. In order to obtain the clues and to dissect the potential molecular mechanisms causing the toxicity, proteomic analysis of SiO_2 _nanoparticle-treated HaCaT cells was carried out in this study.

### Altered protein expression by SiO_2 _exposure

The 16 protein identified by MS reflected a complex cellular response to nano-SiO_2 _exposure. The data showed that the levels of the differentially expressed proteins were associated with the particle size. For most differentially expressed proteins induced by SiO_2 _exposure, the alterations of protein expression were more apparent in 15-nm SiO_2_-treated cells than that in 30-nm or micro-sized SiO_2_-treated cells. The function and the change of expression of these differentially expressed proteins provided insights into the toxic mechanisms of nano-SiO_2 _exposure at protein levels. To better understand the significance of these protein alterations and to evaluate the toxicological relevance, we categorized the proteins listed in Table 2 according to the functions of these proteins.

### Oxidative stress-associated proteins

The major proteins identified in this study are oxidative stress-associated proteins. The change of these proteins indicated oxidative stress could be induced by nano-SiO_2 _exposure, and then initiated the toxic effects. Peroxiredoxins (Prxs) are a ubiquitous family of antioxidant enzymes that control cytokine-induced peroxide levels and thereby mediate signal transduction in mammalian cells [10]. The observed down-regulation of Prx1 and Prx6 in HaCaT cells exposed to 15-nm, 30-nm SiO_2 _and micro-sized SiO_2 _particles indicated that the antioxidant capacity of the cells might be reduced, resulting in the induction of cytotoxicity by SiO_2 _exposure. Moreover, the expression levels of Prx1 and Prx6 were negatively correlated with the particle size, indicating the cellular antioxidant capacity was lower and therefore the cells were easier to be damaged in smaller particle-treated cells. These data were consistent with the results that a higher apoptotic rate occurred in 15-nm SiO_2 _particle-treated cells. Thioredoxin (Trx) is an oxidoreductase enzyme acting as an antioxidant [11]. It plays a vital role in promoting cell growth and inhibiting cell apoptosis to maintain the cell homeostasis at a physiological condition [12]. The significant down-regulation of Trx observed indicated that Trx could be involved in apoptosis observed. Taken together, although it was difficult to define the precise roles of each oxidative stress-associated proteins in the nano-SiO_2_-induced toxicity, our data suggested that the abnormal expression of oxidative stress-associated proteins could be an important mechanism contributing to cell growth inhibition and apoptosis caused by nano-SiO_2 _exposure.

### Cytoskeleton-associated proteins

The cytoskeleton plays a role in controlling cell proliferation, cell cycle and apoptosis [13]. Keratins make up the largest subgroup of intermediate filament (IF) proteins. A major function of keratin IFs is to protect the cells from stresses including apoptotic signals that cause cell rupture and death [14]. The up-regulation of keratin 19 and 4 may represent a compensatory stress response of the cells to the injurious irritation of SiO_2 _exposure.

### Molecular chaperones

The molecular chaperone proteins, like cyclophilin and PPIA, were found to be altered in HaCaT cells by SiO_2 _exposure. Molecular chaperones are responsible for protein folding in the cells to stabilize unfolded or partially folded polypeptides, thereby preventing aggregation, and mediate folding to the native state [15]. The alterations of these molecular chaperones might be indicative of endogenous stress conditions, like oxidative stress [16]. The alterations of these proteins were indirect evidence of nano-SiO_2_-induced cytotoxicity.

### Energy metabolism-associated proteins

The changes of energy metabolism-associated proteins by nano-SiO_2 _exposure indicated that cellular metabolism was affected by the exposure. The alteration of enolase 1 variant indicated that cellular mitochondrial oxidative phosphorylation was suppressed. The change of phosphoglycerate mutase (PGAM) indicated that nano-SiO_2 _exposure could affect the progress of glycolysis. These alterations of protein expression implied that cellular metabolism dysfunction could be induced by SiO_2 _exposure and be also involved in the toxic action of the exposure.

### Apoptosis and tumor-associated proteins

Although the alterations of classic apoptotic marker protein expression were not observed, apoptosis and tumor-related protein maspin was found to be altered by nano-SiO_2 _exposure. Maspin is a serpin that acts as a tumor suppressor in a variety of human cancers, including tumors of the breast and prostate [17]. Its over-expression has been shown to modulate tumor cell apoptosis through the regulation of Bcl-2 family proteins [18]. The significant down-regulation indicated that maspin could be also involved in the pro-apoptotic process during nano-SiO_2 _exposure, and that nano-SiO_2 _exposure could have the potential to promote tumorigenesis.

## Conclusions

In summary, our present study demonstrated that nano-SiO_2 _exposure induced cytotoxicity and altered protein expression in HaCaT cells. The toxic effects were closely related to the particle size as well as the dose used. The alterations in protein expression in the presence of nano-SiO_2 _exposure that resulted in the decreased cell viability, cell cycle change and apoptosis provided further evidence of the toxicological effects of these nanoparticles, and provided valuable clues to elucidate the molecular mechanisms underlying the toxicological effects of nano-SiO_2_exposure. The characteristics and functions of the differentially expressed proteins suggested that in addition to affecting cell growth, cell cycle and apoptosis, nano-SiO_2 _exposure may have the potential to promote tumorigenesis. Our study indicated that nano-SiO_2 _could have potential dermal toxicity, although further experiments may be necessary to confirm whether the responses observed *in vitro *occur *in vivo *in future studies.

## Materials and methods

### Chemicals and antibodies

15-nm, 30-nm and micro-sized SiO_2 _were purchased from Wan Jing New Material Co. Ltd (Hangzhou, Zhejiang, China). Human epidermal keratinocyte cell line HaCaT was purchased from China Center for Type Culture Collection (Wuhan, Hubei, China). MEM culture media were purchased from Hyclone Laboratories, Inc. (Logan, UT, USA). Fetal bovine serum (FBS), penicillin-streptomycin for cell culture and trypsin were purchased from Gibco/Invitrogen (Carlsbad, CA, USA). The kits for cell cycle and apoptosis assay were purchased from Nanjing KeyGen Biotech. Co. Ltd (Nanjing, Jiangsu, China). Cell counting kit-8 (CCK-8) was purchased from Dojindo Molecular Technologies, Inc. (Kumamoto, Japan).

CyDye DIGE fluor dye Cy2, Cy3 and Cy5, Dithiothreitol (DTT), pH gradient Immobiline™ DryStrip (pH3-11NL, 13 cm), IPG gel strip covering oil, IPG buffer(pH3-11NL), glycerol, sodium dodecyl sulfate (SDS), Iodoacetamide (IAA), Acrylamide, N, N'-methylenebisacryl-amide, ammonium persulfate (APS), TEMED, glycine, agarose, protease inhibitor mix, nuclease mix, molecular marker, PhastGel blue, deStreak rehydration solution, 2-D Clean-up kit and 2-D Quant kit were purchased from GE Healthcare (Piscataway, NJ, USA). The primary antibodies rabbit anti-Prx 6 and GSTP1, and the horseradish peroxidase (HRP)-conjugated secondary antibody were purchased from Santa Cruz biotechnology, Inc. (Santa Cruz, CA, USA).

### Characterization of SiO_2 _particles

The characterization of nanomaterials is a very important task in the toxicological and eco-toxicological studies. In theory, detailed parameters of each kind of nanomaterials should be characterized before each toxicological testing, making sure to learn well their association with the biological effects. Therefore, we first characterized some key parameters of the three types of SiO_2 _particles before the cytotoxicological and proteomic studies. The distribution of the three types of SiO_2 _particles was tested by use of Nicomp 380/ZLS submicron particle sizer (Particle Sizing Systems, Santa Barbara, CA, USA). The magnitude of the zeta potential gives an indication of the potential stability of the colloidal system. We measured zeta potential of the three types of SiO_2 _particles by using Nicomp 380/ZLS zeta potential analyzer (Particle Sizing Systems, Santa Barbara, CA, USA) as previously described by Fisher et al., [19]. Crystal structure was characterized by Scintag XDS 2000 diffractometer (Scintag, Inc., Cupertino, CA, USA). The purity of the samples was analyzed by use of a Thermo Elemental X7 ICP-MS spectrometer (Thermo Scientific, Waltham, MA, USA).

### Cell culture and the treatment with SiO_2 _particles

In order to make the SiO_2 _particles distributed in the solution as evenly as possible, the samples were processed by sonication before administered into the cells. HaCaT cells were cultured in MEM media containing 10% FBS, 5% carbon dioxide (CO_2_) at 37°C. SiO_2_particles of different concentrations were administered when the cell confluency reached up to 80%, and the cells were treated for 24 h. The final concentrations of SiO_2 _particles were 80 μg/mL, 60 μg/mL, 40 μg/mL, 20 μg/mL, 10 μg/mL, 5 μg/mL, and 2.5 μg/mL. The cell growth was observed under a light microscope.

### Assay of cell viability

CCK-8 was used to assess cell viability. HaCaT cells were plated into a 96-well plate, and the cells were treated by nano-SiO_2 _or micro-sized SiO_2 _for 24 when the cell confluency reached up to 80%. After the treatment, the cells were incubated with CCK-8 for 2 h. After thoroughly mixing, the plate was read at 450 nm for optical density that is directly correlated with the cell quantity. The inhibitory rate of cell growth was calculated from the relative absorbance at 450 nm. The absorbance was measured at 450 nm using a microplate reader (BioTek, Winooski, VT, USA). The reference wavelength is 630 nm. The inhibitory rate (IR) of the cell growth was figured out by the formula provided by the kit. The IC_50_value was therefore determined.

### Cell cycle assay

The DNA content of cells is associated with the distinct phases of the cell cycle (G_0_, G_1_, S, G_2 _and M). Flow cytometric analysis of cellular DNA content can reveal the distribution of cell cycle and the cellular proliferation activity. HaCaT cells were seeded in a six-well culture plate and treated by SiO_2 _particles when the confluency of the cells reached 80%. After a 24-h treatment, the cells were collected, fixed and permeabilized with 75% ice-cold ethanol overnight at 4°C. The cells were then washed with phosphate buffered saline (PBS) and spinned at 2,000 rpm for 5 min. The supernatant was removed. The cells were resuspended in 100 μL RNase A and incubated at 37°C for 10 min, followed by an addition of 100 μL propidium iodide (PI). After incubation at 4°C for 30 min, the cells were analyzed in a flow cytometer (Becton Dickinson, USA) at 488 nm excitation and 530 nm emission.

### Cellular apoptosis detection

The apoptosis of HaCaT cells was measured as described in the instruction provided by the Annexin V-FITC apoptosis detection kit. In brief, after a 24-h treatment, about 1~5 × 10^5 ^cells were collected and washed with PBS (centrifuged at 2,000 rpm for 5 min). The cells were resuspended in 500 μL binding buffer, and then added by 5 μL of Annexin V-FITC and 5 μL of PI, and incubated in darkness at room temperature for 5~15 min. The cells were analyzed in a flow cytometer.

### 2D-DIGE

#### Protein preparation and protein labeling

After a 24-h treatment with SiO_2 _particles, the cells were collected and lysed on ice for 30 min with lysis buffer (7 mol/L urea, 2 mol/L thiourea, 4% CHAPS, 10 mmol/L Tris). The lysate was harvested into a 1.5 mL centrifuge tube and centrifuged at 12,000 rpm for 30 min at 4°C. Then the supernatant was moved into a new tube, and a part of it was used as source of protein in the following Western blot analysis. The other part was processed with clean up kit. After centrifugation, the pellets were resuspended with solution containing 7 mol/L urea, 2 mol/L thiourea and 2% CHAPS (w/v). Protein concentrations were determined using 2D Quant Kit, and the samples were aliquoted and stored at -70°C until analysis.

The fluorescent dyes labeling for 2D-DIGE was done as previously described [20]. In brief, the lysates from control and treatment groups were labeled with 200 pmol of either Cy3 or Cy5 dyes for comparison on the same gel. The labeling reaction was carried out on ice in the dark for 30 min and then quenched with a 50-fold molar excess of free lysine to dye for 10 min on ice. The internal standard was labeled with Cy2 dye, and this was used as a standard on all gels to aid image matching and cross-gel statistical analysis. The Cy3 and Cy5 labeling reactions from each lysate were mixed and run on the same gels with an equal amount of Cy2-labelled standard. Technical duplicates of 3 independent biological replicates were done in this study.

### Isoelectric focusing (IEF) and SDS-PAGE

A total of 75 μg protein was resolved in 250 μL of rehydration solution containing 7 mol/L urea, 2 mol/L thiourea, 2% CHAPS, 2.8% DTT, 0.5% IPG buffer pH3-11NL and 0.002% bromophenol blue. The protein sample was applied on a 130 mm × 3 mm × 0.5 mm immobilized drystrip (IPG). IEF was performed using the following condition: 12 h at 30 V, 1 h at 500 V, 1 h at 1000 V, until a total of 40,000-50,000 Vhrs at 8,000 V. Each time, four IPG strips were run in parallel. After the first dimension, the strips were equilibrated for 15 min in the buffer containing 6 mol/L urea, 50 mmol/L Tris-HCl pH 8.8, 30% glycerol, 2% SDS, 1% DTT, 0.002% bromophenol blue and then for another 15 min in the similar buffer containing 2.5% iodoacetamide instead of 1% DTT. After equilibration, the strips were loaded onto 12.5% SDS-polyacrylamide constant gel for second dimensional separation. Gels (160 mm × 180 mm × 1.5 mm) were run in an SE600 system (GE Healthcare, Piscataway, NJ, USA) for 15 min at 15 mA per gel and then 25 mA per gel until bromophenol blue reached the bottom of the gel.

### Image acquisition and data analysis

All of the gels were scanned by Typhoon Trio Scanner (GE Healthcare, Piscataway, NJ, USA) to generate gel images from the Cy2, Cy3 and Cy5 labeled samples. The images were cropped using the ImageQuant software tool (GE Healthcare, Piscataway, NJ, USA) and imported into DeCyder™ 2D 6.5 software (GE Healthcare, Piscataway, NJ, USA). The Biological Variation Analysis (BVA) module of Decyder™ 2D 6.5 was used to compare the control with the samples of treatment with SiO_2 _particles to generate lists of differentially expressed proteins.

### Image analysis, spot digestion, and identification of differentially expressed proteins

Preparative gels containing 1 mg of protein were fixed and then post-stained by MS (mass spectrometry)-compatible coomassie stain. The gels were scanned with image scanner (Amersham Biosciences) at 300 pixels per inch (ppi). The gel images were analyzed and matched using Image Master 2D Platinum 5.0 Software (Amersham). Unmatched spots were deserted. The protein spots showing 1.2-fold up/down-regulation or above were considered as differentially expressed proteins. The matched coomassie-stained spots were cut out, destained with a solution of 25 mM ammonium bicarbonate in 50% ACN 30 min at 37°C. Then gel pieces were washed twice with deionized water, shrunk by dehydration in CAN. Samples were swollen in a digestion buffer (20 mmol/L ammonium bicarbonate, 12.5 ng/μL trypsin), after 30-min incubation, the samples were digested for more than 12 h at 37°C. Then peptides were extracted twice using 0.1% TFA in 50% ACN. The extracts were dried under a stream of nitrogen. For MALDI-TOF-MS/MS, the dried samples were redissolved by 0.7 μL 50% acetonitrile, 0.1% trifluoroacetic acid containing 5 mg/mL CHCA. Then the solution was spotted on a stainless steel target with 192 wells (USA). The MALDI mass spectrometer was an ABI 4700 TOF-TOF proteomics analyzer (Applied Biosystems, Framingham, MA, USA) instrument. Myoglobin digested by trypsin was used to calibrate the mass instrument with internal calibration mode. First, MALDI-TOF-MS/MS was performed on all differential spots. Positive ion mass spectra were recorded using 20 kV of total acceleration energy. The mass range was scanned from 700 to 3500 Da. Then the peptides with the highest peak intensity were submitted to MALDI-TOF-TOF analysis. The product ion spectra were searched against the IPI human databases for exact matches using the GPS program (ABI, USA) and the MASCOT search engine http://www.matrixscience.com. Homo sapiens taxonomy restriction was used, and the tolerance of MS/MS was set to ± 0.6 Da.

### Western blot analysis

Equal amounts of protein (30 μg) were isolated in 12.5% sodium dodecyl sulfate-polyacrylamide gel, and blotted onto polyvinylidene difluoride membranes (PVDF; Amersham Pharmacia Biotech, Piscataway, NJ, USA). The membranes were blocked with 5% milk in TBS/0.05% Tween-20 for 1 h at 25°C, and then probed with primary antibodies Prx6 (1:1,000) and GSTP1 (1:1,000). The blots were developed with HRP-conjugated secondary antibody and visualized by enhanced chemiluminescence substrate system (Santa Cruz, CA, USA).

### Statistical analysis

Data were expressed as mean ± SD and analyzed using SPSS 13.0 statistical software (SPSS Inc., Chicago, Illinois, USA). The One-Way ANOVA procedure followed by Student-Newman-Keuls test was used to determine the different means among groups. The level of significance was set at *p *< 0.05.

## Conflict of interest

The authors declare that they have no competing interests.

## Authors' contributions

Authors XFY, JJL, XMW and ZXZ are responsible for the study design and writing of the manuscript. Authors XFY, JJL and HWH are responsible for data and analysis. Authors XFY, HWH, LZ, CMG, LQY, JHY, HYH, LHH and BZ performed the experiments. All the authors read and approved the final manuscript.
